# Impact of LEAP-012 and EMERALD-1 in the management of HCC

**DOI:** 10.1016/j.jhepr.2025.101664

**Published:** 2025-11-06

**Authors:** Amit G. Singal, Kirema Garcia-Reyes, Robin K. Kelley, Edward Kim

**Affiliations:** 1 Department of Internal Medicine, UT Southwestern Medical Center, Dallas TX, United States; 2 Department of Diagnostic, Molecular, and Interventional Radiology, Icahn School of Medicine at Mount Sinai, New York, NY, United States; 3 Division of Hematology/Oncology, Helen Diller Family Comprehensive Cancer Center, University of California, San Francisco, San Francisco, CA, United States

**Keywords:** liver cancer, TACE, combination therapy, immunotherapy

## Abstract

The introduction of immune checkpoint inhibitors (ICIs) for hepatocellular carcinoma (HCC) has spurred interest in evaluating their use in earlier lines of therapy, including in combination with locoregional therapy for patients with intermediate-stage disease. Transarterial chemoembolisation (TACE) is believed to increase neoantigen release and local tumour PD-L1 expression, suggesting the potential for enhanced antitumour responses if combined with ICIs. The EMERALD-1 trial randomised 616 patients with Child-Pugh A-B7 and localised HCC (including 6-8% with Vp1-Vp2 vascular invasion) to durvalumab plus bevacizumab plus TACE, durvalumab plus placebo plus TACE, and placebo plus TACE. The primary endpoint, progression-free survival, was significantly longer with durvalumab plus bevacizumab plus TACE *vs.* TACE alone (hazard ratio [HR] 0.77, 95% CI 0.61–0.98) but not durvalumab plus TACE *vs.* TACE alone (HR 0.94, 95% CI 0.75–1.19). The LEAP-012 trial randomised 480 patients with Child-Pugh A and liver-localised HCC to lenvatinib plus pembrolizumab plus TACE *vs.* placebos plus TACE. Progression-free survival was significantly longer with lenvatinib plus pembrolizumab plus TACE *vs.* TACE alone (HR 0.66, 95% CI 0.51–0.84). Both trials demonstrated increased grade 3-4 treatment-related adverse events in the combination *vs.* TACE alone arms, including those resulting in treatment discontinuation. Early data from LEAP-012 suggested a trend toward overall survival benefit (HR 0.80, 95% CI 0.57–1.11), although this failed to achieve statistical significance on follow-up analyses. Neither trial has yet reported on other outcomes, including quality of life. Clinicians should emphasise individualised patient selection when deciding between TACE plus ICI *vs.* TACE alone in patients with HCC eligible for locoregional therapy.

## Graphical abstract

**Figure:**
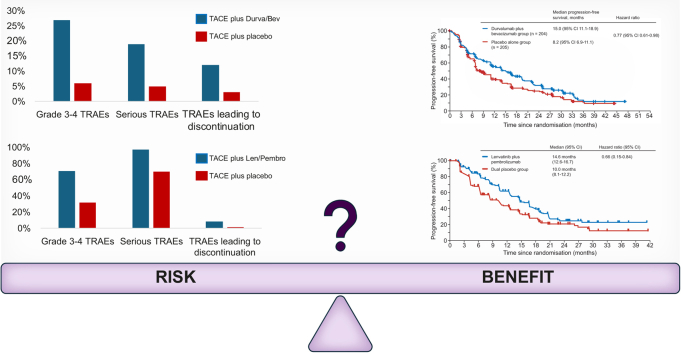

**Keypoints**

• In the EMERALD-1 trial, durvalumab plus bevacizumab plus TACE significantly prolonged progression-free survival compared to TACE alone (HR 0.77, 95% CI 0.61–0.98).
• In the LEAP-012 trial, lenvatinib plus pembrolizumab plus TACE significantly prolonged progression-free survival compared to TACE alone (HR 0.66, 95% CI 0.51–0.84).
• Both trials demonstrated increased grade 3-4 treatment-related adverse events in the combination *vs.* TACE alone arms.
• Current data for overall survival are immature but do not suggest a statistically significant survival benefit for combination therapy *vs.* TACE alone.
• Decisions about using combination TACE plus immune checkpoint inhibitor therapy should be individualised and made in a multidisciplinary manner, considering the totality of clinical data, including safety and patient-reported outcomes.

## Introduction

Hepatocellular carcinoma (HCC) is the third leading cause of cancer-related death globally and one of the few cancers with a 5-year survival rate that has remained below 20%.^1^ Although patients with early-stage HCC can achieve a median survival exceeding 10 years with curative-intent therapies, patients with larger tumour burden are treated with palliative therapies and have a median survival of 2-3 years.^2^ Transarterial therapies including chemoembolisation (TACE) and radioembolisation (TARE) are the recommended therapies for most patients with intermediate-stage HCC that is too extensive for curative therapies.^3^^,^^4^

Recent advances in treatment options, particularly the introduction of immune checkpoint inhibitors (ICIs), have significantly improved prognosis for patients with advanced-stage HCC.^5^ Activity in advanced stages of HCC has spurred interest in evaluating ICIs in earlier lines of therapy, including in patients with intermediate-stage disease. Herein, we review results of the LEAP-012 and EMERALD-1 trials evaluating the combination of ICIs with TACE and discuss how these results can be applied to the management of patients with intermediate-stage HCC.

### Current management of intermediate-stage HCC

The management of intermediate-stage HCC has evolved to incorporate interventional techniques aimed at improving patient outcomes.^6^ TACE has demonstrated safety and efficacy in treating HCC, with improved overall survival (OS) compared to best supportive care.^7^^,^^8^ Based on small randomised controlled trials (RCTs) and meta-analyses, TACE has been a mainstay of therapy for patients who are not candidates for surgical resection or ablation for over two decades and is included in clinical practice guidelines globally.^3^^,^^4^^,^[9], [10], [11] Additionally, TARE has emerged as another effective option. Although there are no adequately powered RCTs demonstrating superiority of TARE *vs.* TACE, several cohort studies have demonstrated increased time to progression (TTP) and comparable OS to TACE.^12^^,^^13^ The TRACE trial (n = 72 patients with intermediate-stage HCC) not only demonstrated improvements in TTP (median 17.1 *vs.* 9.5 months, *p* = 0.002) but also suggested improved OS (median 30.2 *vs.* 15.6 months, *p* = 0.006).^14^ Following the results of the LEGACY study, TARE with Y-90 received FDA approval, further solidifying its role in treatment algorithms and leading to its addition in the most recent Barcelona Clinic Liver Cancer (BCLC) update.^15^ Locoregional therapies have also demonstrated efficacy in patients with limited vascular invasion (Vp1/Vp2) with encouraging downstaging rates. Successful downstaging following locoregional therapy has been linked to post-transplant recurrence rates that are comparable to those of patients who present within the Milan criteria.^16^ However, the best results are observed for patients within UNOS-downstaging criteria and the role of transplant for patients with larger tumour burden, particularly those with vascular invasion at initial presentation, remains controversial.

There is increasing awareness of the heterogeneity in tumour burden, as well as variability in outcomes and prognosis after locoregional therapy, among patients with liver-localised disease. Whereas earlier staging systems, such as that proposed by Okuda and colleagues, stratified intermediate-stage disease according to the degree of liver involvement, the widely adopted BCLC system groups all patients with liver-localised disease beyond the Milan criteria as intermediate-stage HCC.^15^^,^^17^ Subsequent proposals of alternative staging systems, such as the Hong Kong Liver Cancer and Italian Liver Cancer systems, sub-classified patients with BCLC stage B HCC; however, this increased complexity has led to low adoption globally.^18^^,^^19^ More recently, Wang and colleagues published the 6-and-12 score, which is based on the sum of the number of HCC nodules and largest tumour diameter.^20^ Patients undergoing TACE with a score ≤6 had a better OS (median 49.1 months) compared to those with scores >6 but ≤12 (median 32.0 months) and those with scores >12 (median 15.8 months). Similarly, the APPLE (Asia-Pacific Primary Liver Cancer Expert) association has proposed the up-to-7 criteria using a similar method of adding the number of HCC nodules and maximum tumour diameter, based on the premise that patients within up-to-7 criteria have better tolerability and responses to locoregional therapy than those exceeding this threshold.^21^ Moreover, Kudo and colleagues recently published a retrospective propensity score-matched analysis comparing 60 patients with HCC beyond up-to-7 criteria who received TACE as initial therapy compared to 30 patients who received up-front systemic therapy with lenvatinib.^22^ Patients treated with lenvatinib had a higher objective response rate (ORR) (73.3% *vs.* 33.3%) and longer progression-free survival (PFS) (median 16.0 *vs.* 3.0 months), as well as better preservation of liver function, as measured by the albumin-bilirubin (ALBI) score. Notably, two patients were downstaged and underwent curative therapies with local ablation or resection and 10 received subsequent TACE, often in a more selective manner. These interventions were associated with longer OS (median 37.9 *vs.* 21.3 months) in the systemic therapy arm. This “proof of concept” is now being evaluated in large RCTs, including the ABC trial, which is comparing TACE *vs.* atezolizumab plus bevacizumab (NCT04803994) and examining time to failure of the treatment strategy (defined as disease progression with loss of clinical benefit, unacceptable toxicity, liver function deterioration, or inability to continue therapy for other reasons).

### Rationale and results for combination trials with TKIs

TACE produces local ischaemia and a consequent surge in vascular endothelial growth factor (VEGF) levels, which can lead to neo-angiogenesis and TACE failure. Thus, it seems logical that combining TACE with anti-VEGF therapy, such as tyrosine kinase inhibitors (TKIs), could suppress the VEGF surge and improve TACE outcomes. However, several RCTs failed to demonstrate a clinical benefit of adding TKIs to TACE. The SPACE trial was a phase II trial comparing TACE plus sorafenib (n = 154) *vs.* TACE plus placebo (n = 153), with a primary endpoint of TTP.^23^ Median TTP was similar between the two arms (HR 0.80, 95% CI 0.59–1.08), as was OS (HR 0.90, 95% CI 0.61–1.33). Several subsequent RCTs, including Post-TACE and TACE-2, similarly failed to find a benefit of adding sorafenib to TACE on TTP or OS.^24^ The TACTICS trial randomised patients to TACE plus sorafenib (n = 80) or TACE alone (n = 76), with co-primary endpoints of time to unTACEable progression and OS.^25^ Although median time to unTACEable progression was significantly longer in the TACE plus sorafenib arm (HR 0.59, 95% CI 0.41–0.87), there was no significant benefit in OS (HR 0.86, 95% CI 0.61–1.22). In a predefined subgroup analysis, there was a signal for benefit in patients beyond up-to-7 criteria, with a median OS of 25.0 months with TACE alone and 36.3 months with TACE plus sorafenib. Overall, studies have consistently demonstrated a lack of benefit from combining TACE with TKIs, so this approach is not recommended in clinical practice.

## Rationale for combination trials with ICIs

**Fig. 1:**
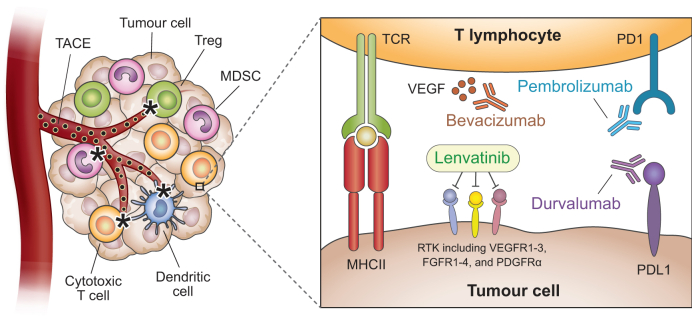
Mechanism of action for combination therapy in EMERALD-1 and LEAP-012. TACE can increase neoantigen release and local tumour PD-L1 expression, as well as reduce peritumoral immune-exhausted T cells and upregulate pro-inflammatory pathways, leading to possible increased antitumour activity when combined with immune checkpoint inhibitor therapy. FGFR, fibroblast growth factor receptor; MDSC, myeloid-derived suppressor cell; PDGFR, platelet-derived growth factor receptor; PD-1, programmed cell death 1; PD-L1, programmed cell death ligand 1; RTK, receptor tyrosine kinase; TACE, transarterial chemoembolisation; TCR, T-cell receptor; VEGFR, vascular endothelial growth factor receptor.

The introduction of ICIs has renewed interest in combination therapy, although the proposed rationale for using them in combination with TACE differs from TKIs (Fig. 1). TACE is thought to increase neoantigen release as well as local tumour programmed cell death ligand 1 (PD-L1) expression, leading to the hypothesis that combinations with anti-programmed cell death 1 (PD-1) or anti-PD-L1 ICIs might increase antitumour responses.^26^ Additionally, TACE may reduce peritumoral immune-exhausted T cells while upregulating pro-inflammatory pathways. However, the mechanisms underlying TACE’s impact on the tumour immune microenvironment are complex and poorly understood.^27^ The preclinical rationale for this combination is supported by prolonged PFS in two landmark RCTs – EMERALD-1 and LEAP-012 – though follow-up is ongoing to determine durability and safety, as detailed below.^28^^,^^29^

### EMERALD-1 trial design and results

The design and outcomes of the EMERALD-1 trial are detailed in Table 1.^28^ In brief, EMERALD-1 included adult patients with HCC not amenable to curative therapies, although it allowed patients to have large tumour burden including Vp1 or Vp2 vascular invasion. Patients were required to have Child-Pugh A or B7 liver function and Eastern Cooperative Oncology Group (ECOG) performance status 0 or 1. Patients (n = 616) were randomized in a 1:1:1 manner to three study arms: durvalumab plus bevacizumab plus TACE, durvalumab plus placebo plus TACE, and two placebos plus TACE. Randomisation was stratified by TACE modality (drug-eluting bead TACE *vs.* conventional TACE), geographic region, and presence of portal vein invasion. The primary endpoint of the trial was PFS, with OS and quality of life as key secondary endpoints. For all three arms, TACE was initiated on day 0 and was allowed up to four times within the first 16 weeks, with the intent of treating all observed tumours. Durvalumab (or placebo) was started during week 1, and bevacizumab (or placebo) was initiated in Arm A after all TACE procedures had been completed.

**Table 1:** Design and outcomes of EMERALD-1 and LEAP-012 trials.

	EMERALD-1 (n = 616)	LEAP-012 (n = 480)
Key inclusion criteria	1. Adult patients with unresectable HCC 2. Amenable to TACE, anticipated to be able to undergo up to 4 TACE procedures 3. Child-Pugh A – B7 and ECOG performance status 0	1. Adult patients with unresectable HCC 2. Amenable to TACE 3. Child-Pugh A, ECOG performance status 0, and hepatoma arterial embolization prognostic score A-C
Key exclusion criteria	1. Prior systemic therapy or TACE 2. Major portal vein invasion (Vp3-4) or metastatic spread	1. Prior systemic therapy or TACE 2. Tumours ≥10 cm in diameter, >10 tumours, or >50% liver involvement 3. Vascular invasion or metastatic spread
Study arms	**Arm A:** Durvalumab plus bevacizumab plus TACE	**Arm A:** Lenvatinib plus pembrolizumab plus TACE
	**Arm B:** Durvalumab plus TACE	
		**Arm B:** Placebo plus TACE
	**Arm C:** Placebo plus TACE	
Stratification variables	1. TACE modality (drug-eluting bead *vs.* conventional) 2. Region (Japan *vs.* rest of Asia *vs.* other regions) 3. Vascular invasion (Vp1/2 *vs.* none)	1. Study site 2. AFP level (≤400 *vs.* >400 ng/ml) 3. ECOG performance status (0 *vs.* 1) 4. ALBI grade (1 *vs.* 2 *vs.* 3) 5. Tumour burden (using 6-and-12 score)
TACE delivery	One to four TACE, all completed within 16 weeks	Maximum of two TACE procedures per tumour
Timing of systemic therapy	1. Durvalumab or placebo at least 7 days after first TACE 2. Concurrent bevacizumab at least 14 days after last TACE	1. Lenvatinib plus pembrolizumab at randomization
Efficacy outcomes∗		
PFS	15.0 *vs.* 8.2 months HR 0.77, 95% CI 0.61–0.98	14.6 *vs.* 10.0 months HR 0.66, 95% CI 0.51–0.84
ORR per RECIST v1.1	44% *vs.* 30%	47% *vs.* 37%
ORR per mRECIST	60% *vs.* 48%	71% *vs.* 50%
OS	Not yet reported	HR 0.80, 95% CI 0.57–1.11
Safety outcomes∗		
Grade 3-4 TRAEs	27% *vs.* 6%	71% *vs.* 32%
TRAEs leading to discontinuation	12% *vs.* 3%	8% *vs.* 1%
TRAEs leading to death	0% *vs.* 1.5%	2% *vs.* <1%
Quality of life	Not yet reported	Not yet reported

AFP, alpha-fetoprotein; ALBI, albumin-bilirubin; ECOG, Eastern Cooperative Oncology Group; HCC, hepatocellular carcinoma; HR, hazard ratio; ORR, objective response rate; OS, overall survival; PFS, progression-free survival; TACE, transarterial chemoembolisation; TRAEs, treatment-related adverse events.

∗ For EMERALD-1 trial, efficacy and safety outcomes were reported for Arm A (durvalumab plus bevacizumab plus TACE) *vs*. Arm C (placebo plus TACE).

Most patients in each arm had one to two TACE procedures, although 15-20% of patients had four TACE procedures. Over 90% of patients in the combination arms received durvalumab, although only 75% of patients in Arm A received bevacizumab. The median age of patients was 65 years, and the majority (>75%) were male. Over 97% of patients in each arm had Child-Pugh A liver disease, and over 82% had ECOG performance status 0. Approximately 25% of patients had BCLC stage A HCC, 55-60% BCLC stage B HCC, and ∼15% BCLC stage C HCC. Similarly, approximately 30% of patients in each arm had a HAP (hepatoma arterial-embolisation prognostic) score of A, ∼35% had a score of B, 20-25% a score of C, and ∼10% a score of D. Over half of patients had tumour burden exceeding up-to-7 criteria, and 6-8% of patients had portal vein invasion.

**Fig. 2:**
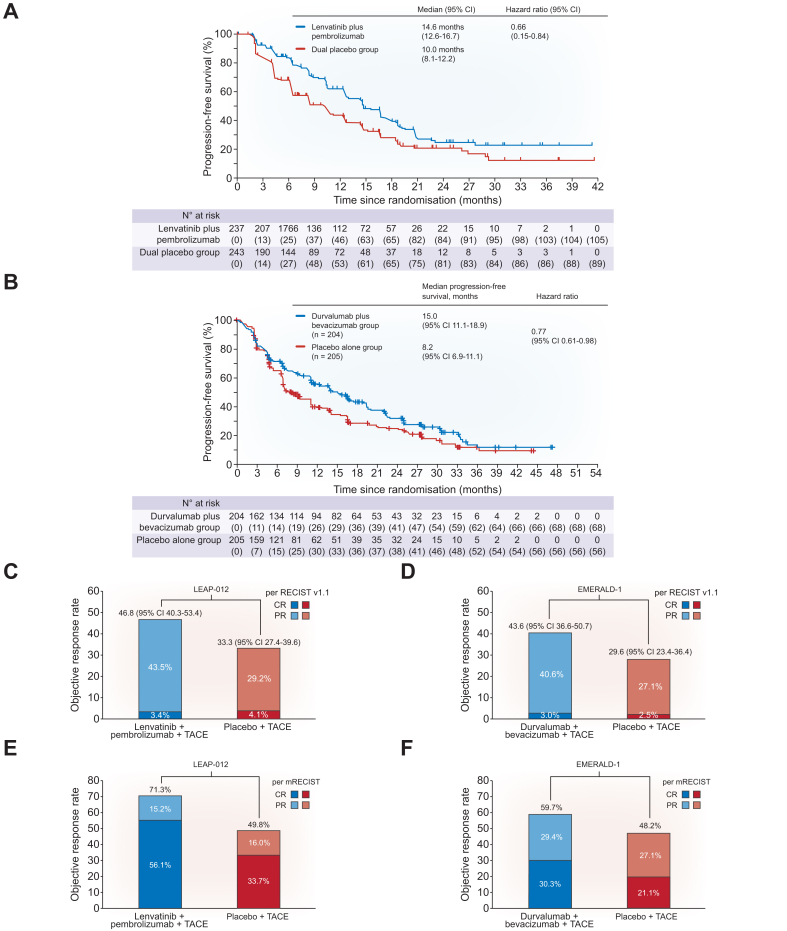
Progression-free survival and objective responses in EMERALD-1 and LEAP-012. Compared to TACE alone, progression-free survival and objective response rates (per RECIST v1.1) were significantly improved in the lenvatinib plus pembrolizumab plus TACE arm (A,C) and the durvalumab plus bevacizumab plus TACE arm (B,D). Similar results were seen with improvements in objective response rates per mRECIST (E,F). CR, complete response; PR, partial response; TACE, transarterial chemoembolisation.

The primary endpoint, PFS, was significantly longer in the durvalumab plus bevacizumab plus TACE arm compared to the TACE alone arm, with median PFS estimates of 15.0 (95% CI 11.1–18.9) and 8.2 (95% CI 6.9–11.1) months, respectively (HR 0.77, 95% CI 0.61–0.98) (Fig. 2).^28^ This improvement was consistent across all examined subgroups, including aetiology of liver disease, baseline tumour burden (within *vs.* beyond up-to-7 criteria), and baseline ALBI score. However, subgroup analyses suggested possible decreased benefit in patients with portal vein invasion, those with ECOG performance status 1, and those with a HAP score of D. Conversely, PFS was not significantly improved in the durvalumab plus TACE arm compared to the TACE alone arm (HR 0.94, 95% CI 0.75–1.19). ORR per RECIST v1.1 was higher in both combination arms compared to the TACE alone arm, with ORRs of 41.0% in the durvalumab plus bevacizumab plus TACE arm and 43.6% in the durvalumab plus TACE arm, compared to 29.8% in the TACE alone arm. ORRs per mRECIST were 59.7% in the durvalumab plus bevacizumab plus TACE arm and 51.7% in the durvalumab plus TACE arm, compared to 48.2% in the TACE alone arm. However, median duration of response appeared longer in the durvalumab plus bevacizumab plus TACE arm than in the durvalumab plus TACE arm (median 22.1 *vs.* 14.0 months). Overall survival data remain immature and have not yet been reported.

There were no unexpected adverse events in any arm, although the combination of durvalumab plus bevacizumab plus TACE was associated with substantially higher rates of grade 3-4 treatment-related adverse events than durvalumab plus TACE and TACE alone (26.6% *vs.* 6.5% and 6.0%, respectively), as well as all-cause adverse events leading to discontinuation (24.7% *vs.* 12.1% and 7.0%, respectively). The most common adverse events in the durvalumab plus bevacizumab plus TACE arm were hypertension, post-embolisation syndrome, hypothyroidism, pruritus, and proteinuria. Treatment-related adverse events leading to death were observed in no patients treated with durvalumab, bevacizumab, nor TACE, compared to 1.5% for TACE plus placebo. Patient-reported outcomes including quality of life measures are awaited.

### LEAP-012 trial design and results

The design and outcomes of the LEAP-012 trial are detailed in Table 1.^29^ The LEAP-012 trial included adult patients with embolisation-eligible, intermediate-stage HCC not amenable to curative therapies. Patients were required to have Child-Pugh A liver function and ECOG performance status 0 or 1. In contrast to EMERALD-1, patients with Child-Pugh B7, 10 or more tumours, tumours ≥10 cm, tumour burden with ≥50% involvement, and those with any vascular invasion were excluded.

Patients (n = 480) were randomized in a 1:1 manner to two study arms: lenvatinib plus pembrolizumab plus TACE *vs.* placebos plus TACE. Randomisation was stratified by study site, alpha-fetoprotein level, ECOG performance status, ALBI grade, and baseline tumour burden (using the 6-and-12 score). The primary endpoints of the trial were PFS and OS, with ORR, duration of response, and quality of life as key secondary endpoints. For both arms, TACE was initiated within 1 month of randomisation and was limited to up to two treatments per tumour, and up to four TACE treatments total. Patients started lenvatinib and pembrolizumab at baseline, with TACE initiation 2-4 weeks after the start of systemic therapy and the duration of pembrolizumab capped at 2 years.

Patients in the lenvatinib plus pembrolizumab plus TACE arm had fewer TACE procedures than those in the TACE alone arm, with 49% and 36%, respectively, undergoing a single TACE procedure. The median age of patients was 66 years, and the majority (>80%) were male. All patients had Child-Pugh A cirrhosis, and ∼90% had ECOG performance status 0. Approximately 30% of patients had BCLC stage A HCC, 55-60% BCLC stage B HCC, and ∼10% BCLC stage C HCC. Nearly half of patients had a tumour burden score (sum of HCC number and maximum diameter) exceeding 6 but only 3% had a tumour burden score exceeding 12.

PFS was significantly longer in the lenvatinib plus pembrolizumab plus TACE arm compared to the TACE alone arm, with median PFS estimates of 14.6 (95% CI 12.6–10.0) and 10.0 (95% CI 8.1–12.2) months, respectively (HR 0.66, 95% CI 0.51–0.84) (Fig. 2).^29^ This improvement was consistent across all examined subgroups, including baseline tumour burden and baseline ALBI score; however, subgroup analyses suggested possible decreased benefit in patients with ECOG performance status 1. ORR per RECIST v1.1 was higher in the combination arm compared to the TACE alone arm, with ORRs of 47% and 33%, respectively. ORR per mRECIST was also higher in the combination arm compared to the TACE alone arm, with ORRs of 71.3% and 49.8%, respectively. Median duration of response appeared longer in the combination arm than the TACE arm (median 12.6 *vs.* 10.7 months). At the first interim analysis, there was a trend toward improved OS (HR 0.80, 95% CI 0.57–1.11). In Kaplan-Meier analysis, the OS curves separated between 6-9 months, with 24-month OS estimates of 75% and 69%, respectively, although the curves appeared to converge again around month 33. However, a subsequent interim analysis failed to demonstrate a significant benefit for OS, and the likelihood of statistical significance in the future was considered low.

Similar to observations in EMERALD-1, lenvatinib plus pembrolizumab plus TACE was associated with a substantially higher rate of grade 3-4 treatment-related adverse events than TACE alone (71% *vs.* 31%), with the most common adverse events being hypertension, thrombocytopenia, increased aspartate aminotransferase or alanine aminotransferase, diarrhoea, and palmar-plantar erythrodysesthesia syndrome. Treatment-related adverse events leading to treatment discontinuation were also more common, occurring in 8.4% of patients in the combination arm compared to 1.2% of patients treated with TACE alone. Treatment-related adverse events leading to death occurred in 1.7% of patients treated with the combination regimen, compared to 0.4% for TACE plus placebo. Data regarding quality of life have not yet been reported.

## Limitations of current data

While the results of LEAP-012 and EMERALD-1 offer promising insights into combination therapy for HCC, several limitations should be considered when interpreting the data.

**Fig. 3:**
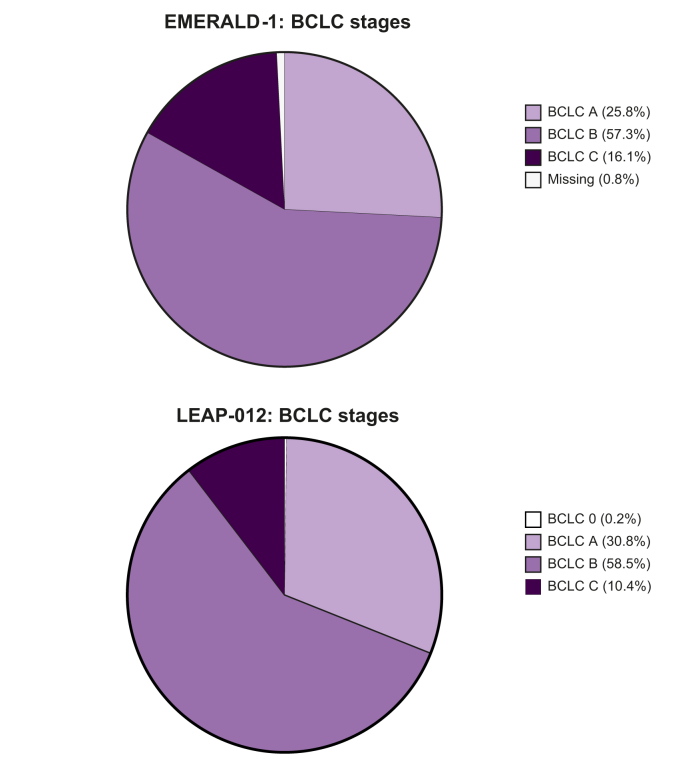
Proportion of patients in EMERALD-1 and LEAP-012 according to BCLC stage. Over 40% of patients in each study had BCLC stage A or stage C disease. BCLC, Barcelona Clinic Liver Cancer.

Both trials enrolled a heterogeneous population, including not only intermediate-stage (BCLC B) patients but also selected patients with early-stage (BCLC A) disease, as well as a subset with advanced-stage (BCLC C) disease, such as those with segmental portal vein invasion or ECOG performance status 1 (Fig. 3). Patients with BCLC stage A HCC typically have a favourable prognosis, so the safety implications and inherent medicalisation required by the investigational regimens may have conferred undue risk without commensurate potential for benefit. Indeed, many of these patients can be downstaged using locoregional approaches alone.^5^^,^^30^ Trials evaluating systemic therapies in the advanced stage setting have similarly enrolled some patients with intermediate-stage HCC who are deemed ineligible for locoregional therapies. Conversely, immunotherapy regimens without TACE have demonstrated survival improvements and are the standard-of-care for patients with BCLC stage C HCC and may offer a better risk-to-benefit ratio than TACE-based combinations. This diversity in baseline characteristics may limit the applicability of findings to well-defined intermediate-stage populations. Whereas EMERALD-1 and LEAP-012 evaluate the incremental benefit of adding systemic therapy to TACE, neither trial addressed whether adding locoregional therapy to systemic therapy improved outcomes for patients with larger tumour burden or limited vascular invasion. Indeed, the absence of a TACE-free arm in either study precluded comparisons of PFS and key endpoints including overall survival and toxicity between the combination approach and systemic therapy alone. This question is particularly important for patients with more advanced burden of disease, in whom TACE may increase the risk of liver dysfunction and liver-related mortality, with less benefit on ORR and HCC-related mortality.

Even among patients with BCLC stage B HCC, both trials did not report granular data regarding tumour burden and location. These data can be important to understand the potential for selective locoregional approaches and the risk of post-treatment liver dysfunction. Although the 6-and-12 score and up-to-7 criteria help distinguish patients with limited *vs.* more extensive intrahepatic disease, they do not account for location of the lesions and number of segments involved. For example, a patient with a unifocal 8 cm exophytic lesion in segment 2 and a patient with five HCC lesions, each 3 cm in maximum diameter, involving different segments would both be regarded as beyond up-to-7; however, the ability to successfully and safely treat the patients with locoregional therapy would likely differ. The former could likely undergo a radiation segmentectomy with favourable outcomes using locoregional therapy alone, whereas the latter patient would likely require multiple treatment sessions and would be at higher risk of resultant liver dysfunction. Similarly, neither trial has reported information on subsequent therapies, including the proportion of patients who were downstaged to curative surgical therapies, including liver transplantation.

In addition, details regarding TACE delivery – including modality (conventional *vs.* drug-eluting bead), embolisation technique, and treatment selectivity – were variably reported. While these technical factors are often cited as sources of heterogeneity, their specific impact on efficacy and tolerability in combination with systemic therapy regimens remains uncertain. Preclinical studies suggest that different TACE modalities may induce distinct immunologic and histopathologic responses, such as variations in tumour hypoxia, acidosis, and immune cell infiltration, with unclear clinical significance.^26^ Selective embolisation may improve tolerability by limiting non-target liver injury and preserving hepatic function, factors that could influence endpoints like PFS and OS.^31^^,^^32^ Despite longstanding calls to standardise TACE techniques, achieving uniformity across diverse global centres is logistically challenging and may not be essential for evaluating the benefits of systemic therapy. Although technique and operator experience may influence outcomes, systematically capturing and controlling for these nuances in large multicentre trials remains difficult. Notably, both EMERALD-1 and LEAP-012 demonstrated relatively consistent results in the TACE only arms despite variations in TACE approach and study design. EMERALD-1 stratified randomisation by the type of TACE (conventional *vs.* drug-eluting bead), whereas LEAP-012 stratified by study site to mitigate centre-specific differences. Rather than attempting to implement strict standardisation, future trials may benefit from more detailed reporting of TACE characteristics – including degree of selectivity and embolisation strategy – to better contextualise differences in outcomes, especially regarding tolerability.

Fourth, the combination regimens were administered in a predefined sequence but differed between the studies. Both lenvatinib and pembrolizumab were started at baseline in the LEAP-012 trial, whereas patients in the triplet therapy arm of EMERALD-1 received durvalumab during week 1 but did not initiate bevacizumab until all TACE procedures had been completed. Delayed bevacizumab initiation was used to address potential concerns of peri-procedural bleeding and increased difficulty of successful embolisation. However, this timing resulted in some patients not receiving bevacizumab as intended and may have theoretically mitigated its observed benefit. As discussed above, the rationale for adding anti-VEGF therapies to TACE is to prevent neovascularisation, which is partly driven by a VEGF surge after the procedure. Prior studies suggest the peak VEGF surge occurs within 1-2 days of TACE, suggesting that greater benefit may be observed if anti-VEGF therapy is initiated prior to the procedure. In contrast to TKIs such as lenvatinib, the long half-life and risk of arterial bleeding with bevacizumab would require a prolonged washout period, rendering dosing prior to TACE infeasible.^33^

Finally, both trials reported significant improvements in PFS; however, it is unclear if this is an accurate surrogate for OS in patients with intermediate-stage HCC. A meta-analysis evaluating ICIs across different cancer types demonstrated that effect sizes for PFS are often larger than those for OS but suggested that a HR for PFS of 0.50 (or lower) may predict an OS benefit.^34^ A similar approach identified a PFS threshold of 0.60 for HCC trials.^35^ However, the validity of PFS likely depends on tumour type and treatment modality, particularly given some immunotherapy regimens may have delayed clinical benefits. Indeed, the utility of PFS as a surrogate for OS is not universally accepted for patients with advanced-stage HCC and has not yet been demonstrated for intermediate-stage disease.^36^ Indeed, it remains unclear whether the observed improvements in PFS reflect a synergistic biological interaction or primarily additive (or even potentially sub-additive) effects. Synergy implies that the combined treatment achieves a benefit greater than the sum of its individual parts, and it is currently unknown whether sequential TACE and systemic therapy would result in the same OS as their concurrent use. This question is particularly important given the higher rates of adverse events observed with combination therapy compared with its individual components, as well as the more limited systemic therapy options available upon progression after combination therapy than after TACE alone. For example, patients treated with TACE plus lenvatinib and pembrolizumab have already been exposed to therapies targeting the anti-PD1/PD-L1 and anti-VEGF pathways. Another challenge inherent to a PFS primary endpoint is the potential for local adjudication of PFS to impact treatment decisions, such as whether to administer additional TACE procedures or discontinue treatment for reported progression based on local PFS assessment, which may subsequently impact the blinded central PFS results. Further, drugs such as lenvatinib and bevacizumab are difficult to effectively “blind” owing to their characteristic adverse event profile, most notably new onset hypertension in most patients, thus introducing a potential for investigator bias in local PFS assessment and treatment decisions. The degree of discordance between local and central PFS assessments has not yet been reported for either study.

These limitations highlight areas for future investigation, including more homogeneous trial eligibility (excluding patients with BCLC stage A and C HCC), standardised reporting of tumour burden and locoregional therapy delivery, better understanding of mechanistic interplay, and biomarker-driven approaches to optimise patient selection and treatment sequencing.

## Applicability to patients treated with TARE

EMERALD-1 and LEAP-012 both evaluated the combination of systemic therapy with TACE; however, TARE has become the most widely used locoregional therapy for intermediate-stage HCC in the United States.^37^ As TARE differs mechanistically from TACE, findings from EMERALD-1 and LEAP-012 may not be directly applicable to patients treated with Y-90. TARE is non-embolic but is instead associated with radiation-induced immunogenic cell death. Studies have suggested an increase in antigen-presenting cells, as well as PD-1 and T-cell immunoglobulins, so adding ICIs may augment the immune-promoting effects of radiation-based therapy.^38^ However, as with TACE, the exact effects of TARE on the tumour microenvironment remain an area of continued investigation.

There are some limited cohort data that have evaluated the combination of ICIs with TARE. The NASIR-HCC trial was an open-label, single-arm phase II study that evaluated the combination of nivolumab plus TARE in 41 patients with large intrahepatic disease or limited vascular invasion.^39^ The authors concluded that the combination was safe, with grade 3-4 treatment-related adverse events in eight patients. However, it was unclear if the combination improved efficacy compared to TARE alone, with an ORR of 41.5%, TTP of 8.8 months, and median OS of 20.9 months. Lee *et al.* also reported results from their SOLID trial, a phase I/IIa trial including 23 patients treated with durvalumab plus TARE for locally advanced unresectable HCC.^40^ The authors concluded that the combination of an anti-PD-L1 agent and TARE was safe, with a TTP of 15.2 months, ORR of 83.3%, and 18-month OS rate of 58.3%. Ongoing trials, such as EMERALD-Y90 (NCT06040099) and ROWAN (NCT05063565), will be critical in defining the role of ICI combinations with TARE.

## Clinical implications

The evolving landscape of HCC treatment, particularly the integration of ICIs with transarterial therapies, presents both opportunities and challenges for clinical practice. While the EMERALD-1 and LEAP-012 trials demonstrated improvements in PFS and higher ORRs for a combination approach than for TACE alone, current data suggest it is unlikely to improve OS. Further follow-up will help determine if similar survival can be observed by using the individual components sequentially after confirmed disease progression, rather than preemptively in all patients. This continued follow-up is particularly important given considerations of potential non-proportional hazards when examining the Kaplan-Meier curve from LEAP-012. Particularly for therapies with high rates of toxicity, cost, or inconvenience, the value of PFS as a surrogate endpoint in oncology requires concordant trends in OS to support its validity. Alternatively, robust improvements in other secondary endpoints such as quality of life scores can also support the value of improvements in PFS. However, high rates of toxicity and treatment discontinuation undermine the of importance of PFS as an in isolation and underscore the need for a comprehensive review of the totality of clinical endpoint data before adoption.^41^

In this context of uncertainty regarding the value of PFS as an isolated endpoint, clinicians must emphasise individualised patient selection and risk assessment when deciding on treatment with TACE plus ICI combination therapy in patients with HCC eligible for locoregional therapy. The heterogeneity of the populations enrolled in these trials highlights the need to tailor therapeutic decisions based on factors such as tumour burden, liver function, performance status, and goals of therapy. Particularly in intermediate-stage disease, endpoints such as PFS, ORR, and quality of life may offer additional actionable insights compared to OS alone. Differences in trial populations, including tumour burden, degree of liver dysfunction, and performance status, also preclude cross-trial comparisons to determine relative efficacy or safety.

In summary, the future of HCC management lies in refining treatment strategies based on tumour biology, liver reserve, and patient-specific goals. While combination therapies represent an exciting frontier, their optimal integration into clinical workflows will require continued evidence generation with adequate lengths of follow-up, as well as multidisciplinary decision-making in clinical practice, considering the totality of clinical data, including safety and patient-reported outcomes.

## Abbreviations

ALBI, albumin-bilirubin; BCLC, Barcelona Clinic Liver Cancer; ECOG, Eastern Cooperative Oncology Group; HCC, hepatocellular carcinoma; ICI, immune checkpoint inhibitors; ORR, objective response rate; OS, overall survival; PD-1, programmed cell death 1; PD-L1, programmed cell death ligand 1; PFS, progression-free survival; RCT, randomised controlled trials; TACE, transarterial chemoembolization; TARE, transarterial radioembolization; TKI, tyrosine kinase inhibitor; TTP, time to progression; VEGF, vascular endothelial growth factor.

## Financial support

Dr. Singal’s research is conducted with support from 10.13039/100000054National Cancer Institute R01 CA256977. The content is solely the responsibility of the authors and does not necessarily represent the official views of the National Institute of Health, Cancer Prevention Research Institute of Texas, or the United States government.

## Authors' contributions

Drafting of manuscript – All authors; Critical revision – All authors.

## Conflicts of interest

Amit Singal has served as a consultant or on advisory boards for Genentech, AstraZeneca, Eisai, Exelixis, Bayer, Merck, Elevar, Boston Scientific, Sirtex, FujiFilm Medical Sciences, Exact Sciences, Roche, ImCare, Curve Biosciences, Glycotest, and Abbott. Kirema Garcia-Reyes has served as a consultant or on advisory boards for Boston Scientific, Cook Medical, Johnson & Johnson, AstraZeneca, Varian, Guerbet. Robin K Kelley reports research funding **to institution** from: Agios, Astra Zeneca, Bayer, BMS, Compass Therapeutics, Eli Lilly, EMD Serono, Exelixis, Genentech/Roche, Loxo Oncology, Merck, Novartis, Partner Therapeutics, QED, Relay Therapeutics, Servier, Surface Oncology, Taiho, Tyra Biosciences; consulting/advisory fees **to institution** from: Agios, Astra Zeneca, BMS, Exelixis, Ipsen, Merck; consulting/advisory fees **to self** from: Astra Zeneca, Compass, CVS Caremark, Elevar, GSK, Jazz, Moderna, Regeneron, Tyra Therapeutics, J-Pharma Inc.; and travel support **to self** from: Astra Zeneca, Merck.
